# Interaction of the *C9orf72*-Amyotrophic Lateral Sclerosis-Related Proline–Arginine Dipeptide Repeat Protein with the RNA-Binding Protein NOVA1 Causes Decreased Expression of UNC13A Due to Enhanced Inclusion of Cryptic Exons, Which Is Reversed by Betulin Treatment

**DOI:** 10.3390/cells12202476

**Published:** 2023-10-18

**Authors:** Ru-Huei Fu, Hui-Jye Chen, Syuan-Yu Hong

**Affiliations:** 1 Graduate Institute of Biomedical Sciences, China Medical University, Taichung 40402, Taiwan; 2 Translational Medicine Research Center, China Medical University Hospital, Taichung 40447, Taiwan; 3 Ph.D. Program for Aging, China Medical University, Taichung 40402, Taiwan; 4 Department of Medicine, School of Medicine, China Medical University, Taichung 40447, Taiwan; 5 Division of Pediatric Neurology, China Medical University Children’s Hospital, Taichung 40447, Taiwan

**Keywords:** amyotrophic lateral sclerosis (ALS), *C9orf72*, proline–arginine dipeptide repeat protein (PR-DPR), UNC13A, cryptic exon (CE), nonsense-mediated mRNA decay (NMD), NOVA1, SH-SY5Y cell, SK-N-DZ cell, betulin

## Abstract

*C9orf72* mutations are the most common form of familial amyotrophic lateral sclerosis (C9-ALS). It causes the production of proline–arginine dipeptide repeat proteins (PR-DPRs) in motor neurons (MNs), leading to the molecular pathology characteristic of ALS. UNC13A is critical for maintaining the synaptic function of MNs. Most ALS patients have nuclear deletion of the splicing repressor TDP-43 in MNs, which causes inclusion of the cryptic exon (CE) of *UNC13A* mRNA, resulting in nonsense-mediated mRNA decay and reduced protein expression. Therefore, in this study, we explored the role of PR-DPR in CE inclusion of *UNC13A* mRNA. Our results showed that PR-DPR (PR_50_) induced CE inclusion and decreased the protein expression of UNC13A in human neuronal cell lines. We also identified an interaction between the RNA-binding protein NOVA1 and PR_50_ by yeast two-hybrid screening. NOVA1 expression is known to be reduced in patients with ALS. We found that knockdown of NOVA1 enhanced CE inclusion of *UNC13A* mRNA. Furthermore, the naturally occurring triterpene betulin can inhibit the interaction between NOVA1 and PR_50_, thus preventing CE inclusion of *UNC13A* mRNA and protein reduction in human neuronal cell lines. This study linked PR-DPR with CE inclusion of *UNC13A* mRNA and developed candidate therapeutic strategies for C9-ALS using betulin.

## 1. Introduction

Amyotrophic lateral sclerosis (ALS) is a rare disease. It causes lethal upper and lower motor neuron (MN) degeneration and skeletal muscle dysregulation in adults. Patients die mainly because of muscle weakness leading to dysphagia and breathing difficulties. At present, there is no effective cure for this disease, and the patients’ survival period is short; therefore, it is urgent to understand its pathogenic mechanism and develop an effective treatment [1]. Studies have shown that approximately 90% of ALS patients are sporadic, which may be related to various environmental factors as well as brain and spinal cord injuries. Approximately 10% are related to genetic variation (familial ALS) [2]. In 90% of all patients with ALS, the characteristics of hyperphosphorylation, cytoplasmic translocation, and decreased nuclear levels of RNA-binding protein TAR-DNA-binding protein 43 (TDP-43) can be found [3].

The familial ALS mutation mainly occurs in the *C9orf72* gene mutation (C9-ALS). A large number of G_4_C_2_ hexanucleotide repeat expansions (HRE) appear inside intron 1 [4]. HRE may directly affect the expression of the *C9orf72* gene (loss of function). It is also possible to produce abnormal repetitive sequences of RNA molecules and proteins (gain of function) [4]. HREs are known to generate five different dipeptide repeat proteins (DPRs) through a repeat-associated non-ATG (RAN) translation mechanism. Among them, proline-arginine-DPR (PR-DPR) is the most toxic [5]. PR-DPR primarily enters the nucleus. Studies have shown that it can damage ribosome biogenesis, nonsense-mediated mRNA decay, stress-induced RNA editing, and RNA-binding proteins (including TAR DNA-binding protein 43) in the nucleus of motor neurons. Likewise, it destroys double-stranded DNA repair, cargo loading, and nuclear import/export. Outside the nucleus, protein translation, neurotransmitter release and recycling, ion channels, ER stress, proteasomes, chaperones, microtubules, the cytoskeleton, and focal adhesion are also affected [6,7,8,9,10,11,12,13,14]. Our previous study showed that PR-DPR can also induce the activity of the NLRP3 inflammasome in microglial cells by inhibiting the function of complement component 1 Q subcomponent-binding protein (C1QBP) [6].

Studies have shown that cytoplasmic aggregation and nuclear depletion of TAR-DNA binding protein 43 (TDP-43) is present in more than 97% cases of ALS [15]. TDP-43 is mainly located in the nucleus and has multiple functions, one of which is as a splicing repressor [16]. When the inhibitory function of TDP-43 is lost in the nucleus, some sequences in the non-conserved intron in the pre-mRNA of a specific gene will be mistakenly included in the mature mRNA to form a cryptic exon (CE). CEs can sometimes cause premature stop codons, transcript degradation, early polyadenylation, reading frame shifts, or changes in RNA stability [17,18]. Genes whose nuclear deletion of TDP-43 induces CEs include Unc-13 homolog A (*UNC13A*), *RAP1GAP*, *AGRN*, *STMN2*, and *PFKP*, which generated at least 179 CEs. Only *UNC13A* located on chromosome 19 has been classified as an ALS risk gene [19,20,21].

**Figure 1:**
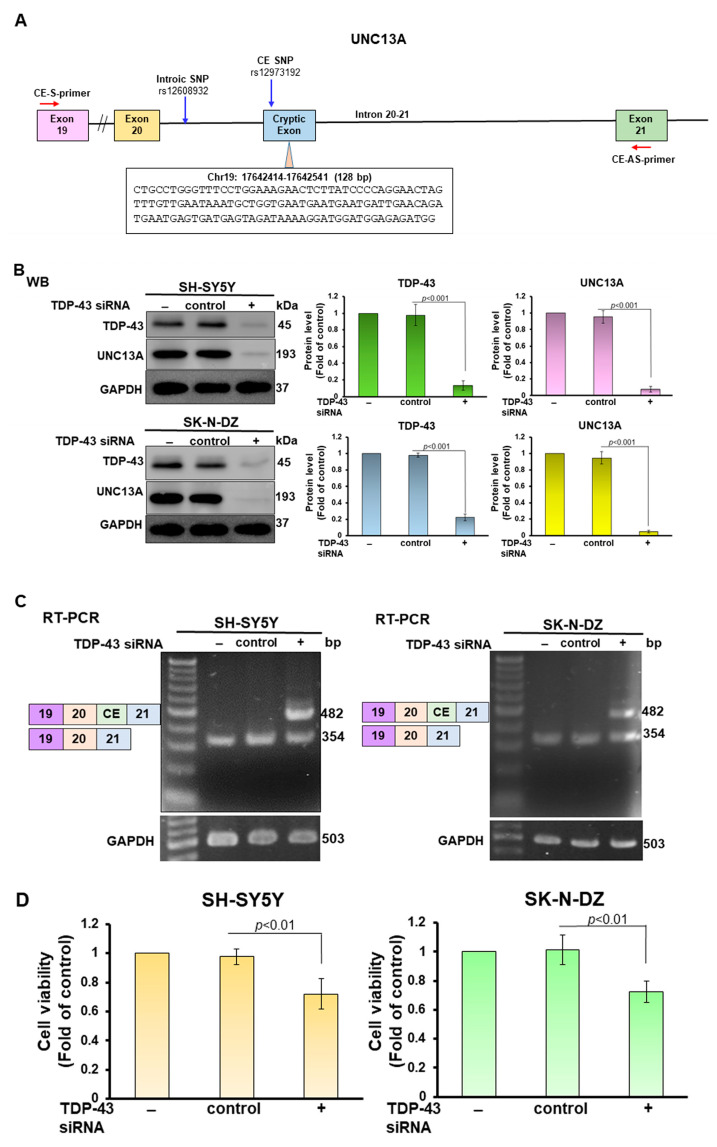

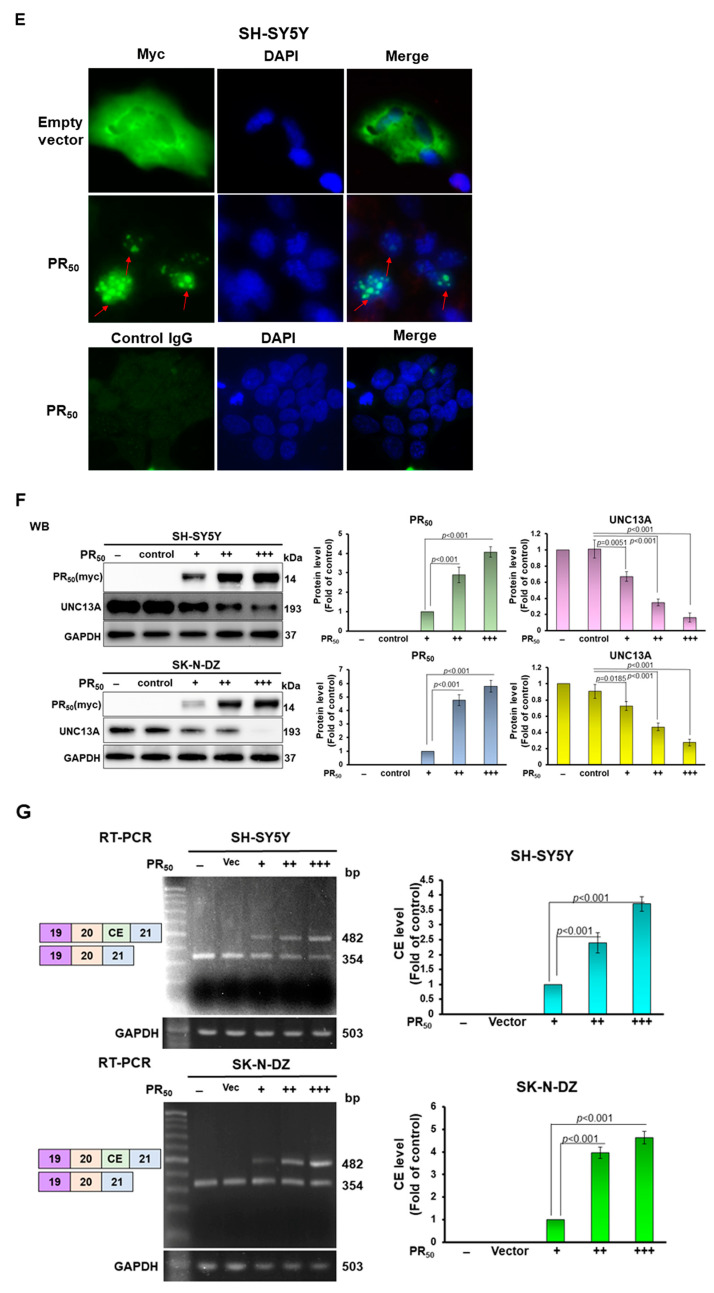

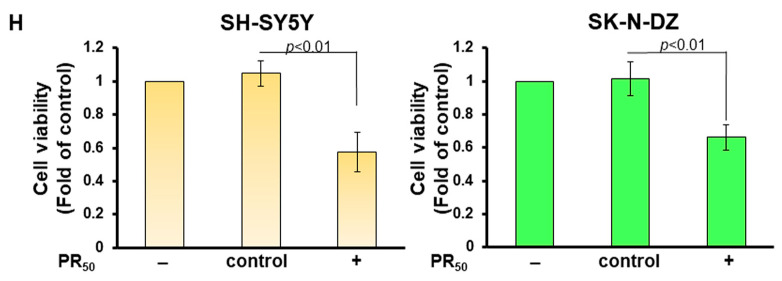
Expression of PR-DPR in human SH-SY5Y and SK-N-DZ neural cell lines can induce the inclusion of cryptic exons (CEs) in UNC13A mRNA, leading to downregulation of protein expression. (**A**) Schematic representation of the CE structure of human *UNC13A* mRNA. The CE is mainly located in the intron between exon 20 and exon 21. It contains 128 nucleotides. Several SNPs in ALS are known to affect CE formation, and their locations are indicated by blue arrows. The red arrows are the relative positions of the primer pairs used to confirm the presence of CE. (**B**) TDP-43 was knocked down in SH-SY5Y and SK-N-DZ cell lines by siRNA, and the expression of TDP-43 and UNC13A proteins was analyzed by Western blotting. (**C**) RNA was extracted from the cell line of (**B**), and RT-PCR was performed with a specific primer pair to confirm the presence of the CE in *UNC13A* mRNA. A common 354 bp fragment can be observed under DNA electrophoresis, and a 482 bp fragment will appear if a CE is included. (**D**) Determining the survival rate of TDP-43 knockdown cells using MTT assay. (**E**) Immunofluorescent staining of PR_50_ (green). DAPI-stained nuclei. The results showed that PR_50_ was mainly distributed in the nuclei and formed dots. (**F**) The PR_50_-expressing SH-SY5Y and SK-N-DZ cell lines were analyzed by Western blotting for the expression of PR_50_ (Myc) and UNC13A. + signs indicate the level of PR_50_ expression. (**G**) RNA was extracted from the cell line of (**F**) and subjected to RT-PCR to confirm the CE inclusion of *UNC13A* mRNA. + signs indicate the level of PR_50_ expression. The results showed that PR_50_ expression induced CE inclusion of *UNC13A* mRNA. (**H**) Determining the survival rate of PR_50_-expressing cells using MTT assay. The above analysis of protein expression and DNA quantity used GAPDH as the internal loading control. In addition, the signal intensity was quantified using ImageJ software (version 1.53).

UNC13A is an essential neuronal protein. It is conserved in primates. However, it is not conserved in mice and is mainly located at synapses in the central nervous system and neuromuscular junctions. It plays a key role in calcium-triggered synaptic vesicle fusion, maturation, initiation, and release [22]. In mouse models, UNC13A is mostly localized to the synapses of glutamatergic neurons in the hippocampus. Action-potential-induced neurotransmitter release at these synapses can be blocked by *UNC13A* knockout [23]. Furthermore, loss of *UNC13A* impairs transmission at the neuromuscular junction [24]. A genome-wide association study (GWAS) has shown that single nucleotide polymorphism (SNP) at specific loci in *UNC13A* increases ALS risk, including susceptibility to ALS and decreased patient survival [25]. Studies have shown that deletion of TDP-43 in the nucleus induces the inclusion of a CE between exons 20 and 21 in *UNC13A* mature mRNA (Figure 1A). This scenario causes a change in the reading frame of *UNC13A* gene expression, resulting in nonsense-mediated decay (NMD) and reduced protein levels [19,26]. Several *UNC13A*-associated SNPs linked with ALS risk were located within (rs12973192) or near CEs (rs12608932) and overlapped with the TDP-43 binding site (Figure 1A) [19,26,27]. Therefore, TDP-43 is crucial for maintaining the correct mRNA splicing of *UNC13A*, thereby maintaining the normal expression of UNC13A and synaptic function. Koike et al. found that TDP-43 inhibits the CE inclusion of *UNC13A* Mrna mainly through the N terminus. It was also confirmed that RNA-binding proteins such as hnRNP L, hnRNP A1, and hnRNP A2B1 also inhibit CE inclusion of *UNC13A* mRNA independently of TDP-43 [28].

Previous reports have indicated that CE inclusion of *UNC13A* mRNA was also found in some C9-ALS patients. PR-DPR is one of the important pathological features of C9-ALS. its primary distribution is in the cell nucleus and influences multiple RNA-binding protein activities. Therefore, in this study, we investigated the association of PR-DPR expression in neurons with CE inclusion in *UNC13A* mRNA and its possible mechanism. First, we used SH-SY5Y and SK-N-DZ neuronal cell lines as the research objects according to previous research methods. The results showed that PR-DPR expression was mainly in the nucleus and significantly caused the inclusion of a *UNC13A* mRNA CE, resulting in a decrease in UNC13A protein expression. To further understand the possible mechanism of this phenomenon, we used yeast two-hybrid screening to find possible PR-DPR interaction partners. Interestingly, we did not find TDP-43 among the candidate positive clones. However, another splicing factor, NOVA alternative splicing regulator 1 (NOVA1), was identified. NOVA1 is a neuron-specific RNA-binding protein that is exclusively expressed in the brain and can inhibit and promote specific splicing events by binding to pre-mRNA [29].

The study revealed that the abnormal function of NOVA1 is linked to alterations in alternative splicing (AS) in early ALS [29]. NOVA1 forms insoluble aggregates in motor neurons derived from induced pluripotent stem cell lines from sporadic and familial ALS patients. Many ALS-associated AS events also contain possible binding sites of NOVA1 [30]. In our study, knockdown of the expression of NOVA1 can significantly enhance the CE inclusion of UNC13A mRNA and downregulate UNC13A protein expression. This result indicated that PR-DPR may promote CE inclusion of *UNC13A* mRNA partly by inhibiting the function of NOVA1. Based on this result, we also found that betulin treatment inhibited the interaction of PR-DPR with NOVA1. It partially reversed the increase in CE inclusion of UNC13A mRNA and the decrease in protein levels induced by PR-DPR. Therefore, BT deserves further evaluation for its utility in the treatment of C9-ALS.

## 2. Materials and Methods

### 2.1. Chemicals, Media, and Neuron-like Cell Line Maintenance

All chemicals used in this study were purchased from Sigma-Aldrich (St. Louis, MO, USA) unless otherwise stated. Cell culture medium, fetal bovine serum (FBS), and related reagents were purchased from Gibco, ThermoFisher Scientific (Waltham, MA, USA). The SH-SY5Y cell line (CRL2266, American Type Culture Collection (ATCC), Manassas, VA, USA) was provided by Dr. Chia-Wen Tsai of China Medical University (Taichung, Taiwan). SK-N-DZ (CRL2149) was obtained from the American Type Culture Collection (ATCC). SH-SY5Y cells were cultured in DMEM supplemented with L-glutamine (2 mM), sodium pyruvate (1.0 mM), sodium bicarbonate (1.5 g/L), nonessential amino acids (0.1 mM), penicillin (1 × 10^5^ units/L)/streptomycin (100 mg/L), and fetal bovine serum (FBS, 10%). SK-N-DZ cells were cultured in RPMI 1640 medium supplemented with L-glutamine, penicillin/streptomycin, and FBS. Both cell lines were maintained at 37 °C under a humidified atmosphere of 95% air and 5% CO_2_.

### 2.2. Processing of Small RNA Interference in SH-SY5Y and SK-N-DZ Cells

Small interfering RNA (siRNA; 75 nM) from *TDP-43*, *NOVA1*, or non-targeting control was transfected using Lipofectamine 2000 Transfection Reagent (Invitrogen, Carlsbad, CA, USA). All steps were performed according to the manufacturer’s protocol. Briefly, cells were seeded in 6-well culture plates (2.0 × 10^5^ cells/per well). When 70% confluence was reached, siRNA transfection proceeded for 24 h. Then, Western blot and RT-PCR analyses were performed. The sequence of siTDP-43 is 5′-UGAGCCCAUUGAAAUACCAUCGGAA-3′. The sequence of siNOVA1 is 5-UGCAACUGAACAAUUGUCU-3′. The sequence of the negative control is 5′-ACGUGACACGUUCGGAGAATT-3′.

### 2.3. Western Blotting of SH-SY5Y and SK-N-DZ Cells

Cell lysates were prepared using RIPA Lysis and Extraction Buffer (ThermoFisher Scientific) containing Halt Protease and Phosphatase Inhibitors Cocktail (ThermoFisher Scientific). Proteins in lysates were quantified using the RC DC Protein Assay Kit (Bio-Rad Life Science, Hercules, CA, USA). Fifty micrograms of the lysate were subjected to sodium dodecyl sulfate-polyacrylamide gel electrophoresis (10–12.5%, Amresco, Solon, OH, USA), and separated proteins were then transferred to PVDF membranes (Millipore Corp., Burlington, MA, USA). Next, the membrane was reacted with TDP-43 (Cell Signaling Technology, Beverly, MA, USA), UNC13A (ThermoFisher Scientific), GAPDH (Cell Signaling Technology), Myc (Cell Signaling Technology), or NOVA1 (Sigma-Aldrich) antibodies at 4 °C overnight. The next day, HRP-conjugated goat anti-mouse or goat anti-rabbit antibodies (Enzo Life Sciences, Farmingdale, NY, USA) were incubated with the membranes for 1 h at room temperature. Finally, the location and level of specific proteins were visualized using an Amersham enhanced chemiluminescence system (Piscataway, NJ, USA). Luminescence signals were detected using the UVP BioSpectrum Imaging System (Upland, CA, USA).

### 2.4. Total RNA Extraction and Reverse Transcriptase-Polymerase Chain Reaction (RT-PCR) of SH-SY5Y and SK-N-DZ Cells

We isolated total RNA from cells using TRIzol reagent (Invitrogen, Carlsbad, CA, USA) according to the manufacturer’s instructions. Next, we identified the CE inclusion of *UNC13A* mRNA using the SuperScript One-Step Reverse Transcriptase-Polymerase Chain Reaction (RT-PCR) system (Invitrogen). In brief, the mRNA reaction mixture was incubated at 55 °C for 60 min to synthesize cDNA. Then, the PCR reaction was performed according to the following steps: the first cycle was 94 °C for 2 min; then, 35 cycles were performed at 94 °C for 15 s, 60 °C for 30 s, and 68 °C for 30 s; the last cycle was at 5 min at 68 °C. The primer sequences for detecting CE inclusion were 5′-TGTCACAATTCCTCCGACCG-3′ (forward) and 5′-ATCGTCACCCTTGGCATCTG-3′ (reverse) (Tri-i Biotech, Taipei, Taiwan). GAPDH was used as a loading control. Its sequence is 5′-GGGAGCCAAAAGGGTCATCA-3′ (forward) and 5′-CCACCTGGTGCTCAGTGTAG-3′ (reverse) (Tri-i Biotech). Amplified products were separated by agarose gel electrophoresis and visualized by ethidium bromide staining.

### 2.5. Recombinant Plasmid Construction and Transfection

The PR-DPR(PR_50_) DNA, *UNC13A* gene fragment (exon20 to exon21 including CE included intron), and the coding cDNA of *NOVA1* were synthesized by Genomics (Taipei, Taiwan). PR_50_ was inserted into the pcDNA 3.1/myc-His vector (Invitrogen) after restriction enzyme digestion. Next, we transiently transfected the expression vectors into SH-SY5Y and SK-N-DZ cells using Lipofectamine 2000 transfection reagent (Invitrogen) according to the manufacturer’s instructions and then selected in Geneticin (G418) (Invitrogen). The successfully transfected population was expanded for further experiments.

### 2.6. Yeast Two-Hybrid Screening of PR_50_-Associated Protein

We used the Matchmaker GAL4 yeast two-hybrid system (Clontech, Mountain View, CA, USA) as described in the product manual. PR_50_ was cloned into the pGBKT7 vector (containing the GAL4 DNA-binding domain DNA-BD) as bait and pre-transformed into the S. cerevisiae host strain of AH109. A cDNA library (Clontech) of the human brain was constructed on the pGADT7 vector (containing the GAL4 activation domain AD) and expressed in the host strain of Y187 as prey. The Y187 library strain was then mated with the PR_50_-expressing AH109 strain to generate diploids. Transcription of *ADE2*, *HIS3*, *MEL1*, and *LacZ* reporter genes is activated if DNA-BD and AD in diploids interact via bait and prey fusion proteins to form intact GAL4 transcription factors. This is reflected in blue positive diploids on SD/-Ade/-His/-Leu/-Trp/X-α-gal plates. To identify the cDNA fragments of the prey contained in these positive diploids, we used the Zymoprep™ Yeast Plasmid Miniprep I (Zymo Research Corporation, Irvine, CA, USA) kit to recover prey plasmids from positive diploids on SD/-Ade/-His/-Leu cultures. The plasmids were then sequenced and analyzed.

### 2.7. Yeast Two-Hybrid Assay for PR_50_ and NOVA1

The PR_50_ DNA sequence was subcloned into the pGBKT7 vector and transformed into the yeast strain AH109. At the same time, the full-length and partial fragments of the coding cDNA of *NOVA1* were subcloned into the pGADT7 vector and transformed into yeast strain Y187. Yeast two-hybrid assays were then performed as described in Section 2.6. In addition, the yeast-two-hybrid assay in which PR_50_ and NOVA1 were exchanged in expression vectors was performed. Diploid yeast co-expressed with pGBKT7-p53 and pGADT7-T was used as a positive control group.

### 2.8. Co-Immunoprecipitation Analysis of 293T Cells

First, PR_50_ DNA was subcloned into the pCMV-HA vector (Clontech), and *NOVA1*-coding cDNA was subcloned into the pCMV-Myc vector (Clontech). The plasmids were then co-transfected into 293T cells using Lipofectamine 2000 (Invitrogen). After 24 h, the cells were lysed and supernatants were collected. For co-immunoprecipitation, we first purified the supernatant with Protein G-Sepharose beads and then immunoprecipitated it with rabbit anti-HA tag antibody (Cell Signaling Technology) or normal rabbit immunoglobulin G (Cell Signaling Technology) at 4 °C for 2 h, followed by incubation of Protein G-Sepharose beads for 1 h. Finally, the immunoprecipitated complexes were washed and subjected to Western blotting. NOVA1 and PR_50_ proteins were detected using mouse anti-Myc and mouse anti-HA tag antibodies (Cell Signaling Technology), respectively. In addition, the vectors of PR_50_ and NOVA1 were exchanged to perform the above experiments.

### 2.9. Immunofluorescence Staining of PR_50_-Expressing SH-SY5Y Cells

SH-SY5Y cells expressing PR_50_ on coverslips were fixed with 4% paraformaldehyde and permeabilized with 0.2% Triton X-100. The coverslips were then soaked in blocking solution (1% bovine serum albumin and 22.52 mg/mL glycine in PBST) for 30 min. Then, mouse anti-Myc (1:500) and rabbit anti-NOVA1 antibodies (1:200) were added and incubated overnight at 4 °C. The next day, the coverslips were washed and placed in blocking solution containing Alexa Fluor 488-conjugated goat anti-mouse secondary antibody (Invitrogen) and Alexa Fluor 568-conjugated goat anti-rabbit secondary antibody (Invitrogen) at 25 °C for 1 h. Finally, the coverslips were washed and the nuclei were stained with 4,6-diamidino-2-phenylindole (DAPI). Fluorescence signals were detected using a Zeiss Axio Imager A1 fluorescence microscope (Carl Zeiss MicroImaging GmbH, Göttingen, Germany).

### 2.10. Screening Inhibitors of the Interaction between GA_50_ and NOVA1 Using a Yeast Two-Hybrid-Based Growth Assay

Synthetic betulin (mol. wt. 442.7, 98% purity, Rainbow Biotechnology Co., Ltd., Shilin, Taipei, Taiwan) was prepared as 10 mM stock solution (in DMSO). Diploid yeast expressing BD-/AD-, BD-p53/AD-T, BD-PR_50_/AD-N3 (NOVA1), or BD-N3/AD-PR50 were cultured in non-selective broth (SD/-Leu/-Trp) containing DMSO, 0, 1, 2, or 4 μM betulin to logarithmic or mid-log phase (30 °C), respectively. Next, the experiment was divided into the following two groups. In the yeast spot assay, the concentrations of diploid yeast in each group were normalized and serially diluted. The yeast cultures (10 μL) were pipetted onto non-selective plates (SD/-Leu/-Trp) or selective plates (SD/-Ade/-His/-Leu/-Trp) to grow at 30 °C for 3 days. The growth of the spot was observed with the naked eye. For absorbance measurements, groups of diploid yeast were normalized and grown for two days in selective or non-selective broth containing 0, 1, 2, or 4 μM betulin. OD values were recorded every 12 h.

### 2.11. Treatment with Betulin and Cell Viability Analysis of SH-SY5Y and SK-N-DZ Cells

On BT treatment, we replaced PR_50_-expressing SH-SY5Y and SK-N-DZ cells with fresh medium and treated them with serial dilutions of BT for 24 h, then collected cells for subsequent experiments. The toxicity of BT was confirmed using CellTiter-Blue^®^ Reagent (Promega, Madison, WI, USA). The reagent was added directly to the culture medium for 2 h at 37 °C. Then, a SpectraMax M2 reader (Molecular Devices, Silicon Valley, CA, USA) was used to quantify the viability of the cells by fluorescent signal intensity (excitation: 560 nm, emission: 590 nm).

### 2.12. Statistical Methods Used in This Study

Each research work in this study was performed in triplicate. We employed SAS software 9.3 (SAS, Institute. Inc., Cary, NC, USA) for statistical processing of general data. Researched values are presented as mean ± standard deviation (SD). Statistical significance was shown with a *p*-value <0.05 and obtained by using one-way analysis of variance (ANOVA) and Tukey’s test. Comparisons between two groups were implemented using Student’s *t*-test.

## 3. Results

### 3.1. PR-DPR Expression Promotes the Inclusion of Cryptic Exon (CE) in UNC13A mRNA in Neuronal Cell Lines, Resulting in a Decrease in the Expression of UNC13A Protein

The structural map of the TDP-43-associated CE of human *UNC13A* mRNA is shown in Figure 1A. The CE contains 128 nucleotides. Previous studies have shown that CE inclusion of *UNC13A* mRNA induced by TDP-43 deletion can be observed in SH-SY5Y and SK-N-DZ neuronal cell models [19]. Therefore, we first blocked TDP-43 expression by RNAi in SH-SY5Y and SK-N-DZ cells. The results showed that TDP-43 knockdown significantly decreased the expression of UNC13A protein in both cell lines compared with the control siRNA group (*p* < 0.001, Figure 1B). Then, RT-PCR was performed using the primer pairs designed for exon 19 and exon 21 to detect CEs (Figure 1A). DNA electrophoresis showed that knockdown of TDP-43 expression resulted in a 482 bp fragment containing the CE in addition to the common 354 bp fragment (Figure 1C). In addition, we colonized and sequenced a fragment containing 482 bp and confirmed that it contained the CE sequence. This result is consistent with the research results of Brown [19] and Ma [26] et al. We also found that *TDP-43* knockdown decreased cell survival rate (*p* < 0.01, Figure 1D). Next, we expressed PR-DPR (PR_50_) in SH-SY5Y and SK-N-DZ cell lines (Figure 1E). Immunofluorescence staining showed that PR_50_ was mainly located in the nuclei to form punctates. Western blotting showed that the expression of PR_50_ would reduce the level of UNC13A protein in cells (*p* < 0.001, Figure 1F). RT-PCR showed that PR_50_ could significantly induce CE inclusion of UNC13A mRNA *(p* < 0.001, Figure 1G). MTT assay revealed that PR_50_ expression decreased cell survival rate (*p* < 0.01, Figure 1H). The above results show that PR-DPR expression in neuronal cell lines causes CE inclusion of UNC13A mRNA as induced by TDP-43 deletion and leads to downregulation of protein expression.

### 3.2. RNA-Binding Protein NOVA1 Is a Specific Interaction Partner of PR-DPR

**Figure 2:**
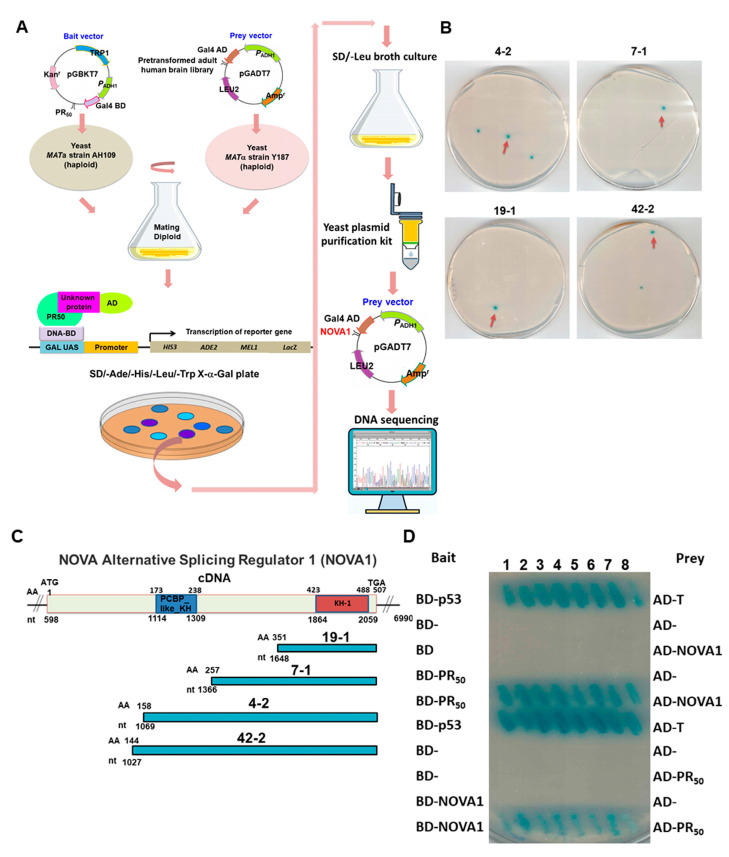

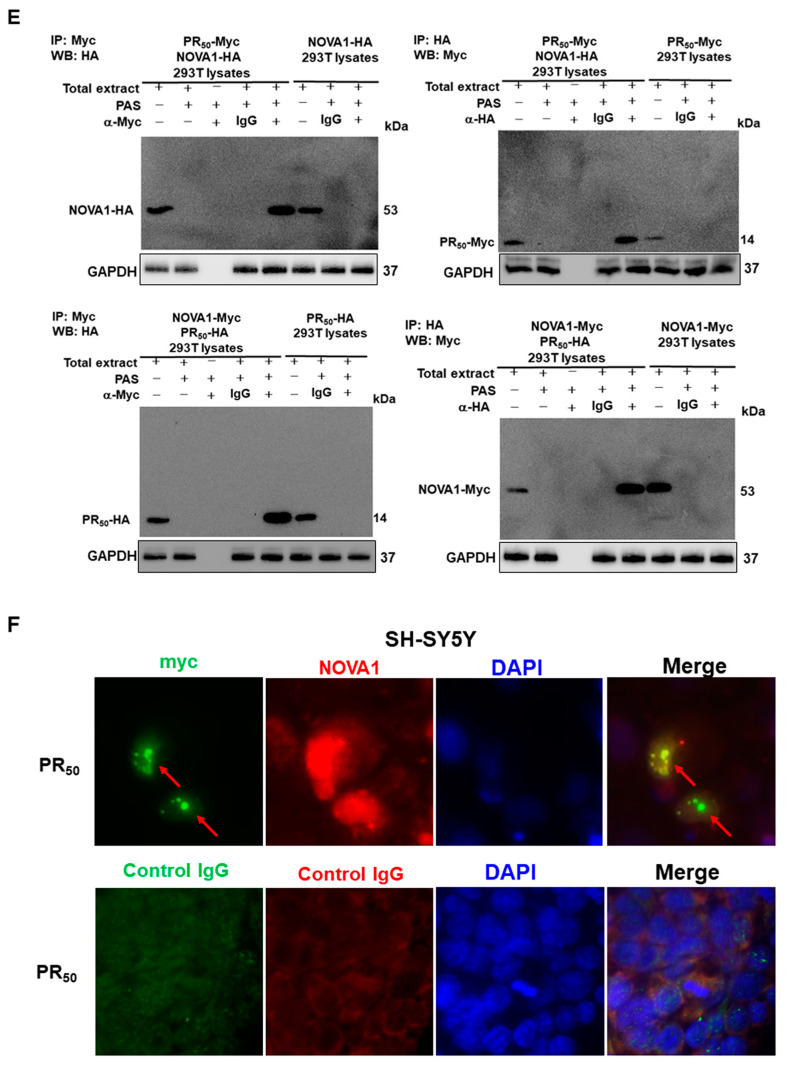
PR-DPR specifically interacts with NOVA1 in yeast, 293T cells, and SH-SY5Y cells. (**A**) Schematic of the yeast two-hybrid screening strategy. Screening of NOVA1 prey clones from an adult brain cDNA library using PR_50_ as bait. The Gal4 transcription factor in diploid cells is activated by interacting two expressed fusion proteins (DNA-BD and AD domain contacts). Finally, activated Gal4 induces the reporter gene expression to establish blue clones on selective plates. (**B**) The positions of four positive clones carrying *NOVA1* cDNA fragments obtained by yeast two-hybrid screening. (**C**) Schematic of the length of the sequenced coding region of the clone carrying the *NOVA1* cDNA fragment (NCBI reference sequence: NM_002515). NOVA1 includes a K homology RNA-binding domain, type I (KH-I) functional region, and a K homology RNA-binding domain, PCBP_like (PCBP_like_KH) functional region. (**D**) Confirmation of direct interaction of PR_50_ with the coding region of full-length NOVA1 cDNA using yeast two-hybrid analysis. Diploid cells expressing both BD-p53 and AD-T antigen were used as positive controls. (**E**) The interaction between PR_50_ and NOVA1 was confirmed by co-immunoprecipitation analysis. We co-expressed Myc-tagged PR_50_ and HA-tagged NOVA1 in 293T cells. Cell extracts were then immunoprecipitated using rabbit anti-Myc (or HA) antibody. Finally, Western blot analysis was performed using a mouse anti-HA (or Myc) antibody. The 293T cells transfected with HA-tagged NOVA1 (or Myc-tagged PR_50_) alone were used as negative controls. We also performed this experiment on 293T cells coexpressing PR_50_-HA and NOVA1-Myc. GAPDH is the equivalent loading control. (**F**) Immunofluorescence staining showed that PR_50_ (green) and NOVA1 (red) were partially colocalized in SH-SY5Y nuclei (DAPI staining, blue).

Previous reports have indicated that PR-DPR can reduce the activity of TDP-43 in cells [31] and induce its mislocalization [32]. This may be a reason leading to the CE inclusion generation of UNC13A observed in this study. To further explore the influence of other possible splicing regulators in this context, we used PR_50_ as bait to screen the human brain cDNA expression library by yeast two-hybrid screening to identify possible interacting target proteins (Figure 2A). Finally, we screened a total of 6.9 × 10^5^ colonies and obtained 84 positive clones. The prey plasmids of four positive clones carried partial cDNA of NOVA alternative splicing regulator 1 (*NOVA1*, NM_002515) (Figure 2B). Among them, the largest positive clone (42-2) contained the *NOVA1* open reading frame encoding 144 to 507 (Figure 2C). Interestingly, we did not obtain any positive clones with TDP-43 cDNA fragments. Next, we performed yeast two-hybrid experiments on PR_50_ and full-length NOVA1 sequences. The results showed that PR_50_ specifically interacted with NOVA1 (Figure 2D). In addition, we co-expressed PR_50_ and NOVA1 in 293T cells for immunoprecipitation analysis. The results showed that PR_50_ and NOVA1 co-localized on the same immunoprecipitated complex (Figure 2E). Furthermore, immunofluorescence staining showed that some PR_50_ and NOVA1 were colocalized in the nucleus (Figure 2F). These results suggest that PR_50_ and NOVA1 interact directly and specifically in the nucleus.

### 3.3. PR-DPR Interacts with the Amino Acid Fragment at the C Terminus of NOVA1

**Figure 3:**
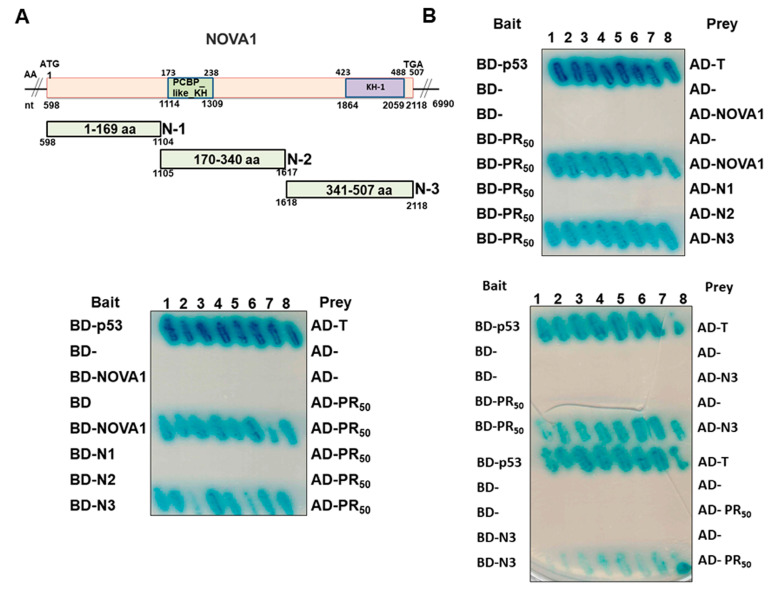
PR-DPR mainly interacts with the C-terminal fragment of NOVA1 in the yeast two-hybrid assay. (**A**) Schematic representation of the three deletion mutants of NOVA1 used in this test. The N1 fragment covers amino acid sequences 1–169, the N2 fragment covers amino acid sequences 170–340, and the N3 fragment covers amino acid sequences 340–507. (**B**) The yeast two-hybrid assay shows that PR_50_ only interacts with the N3 fragment. Diploid yeast expressing both BD-p53 and AD-T antigen were used as positive controls.

To understand the effect of the binding of PR-DPR and NOVA1 on the function of NOVA1, we identified the region where NOVA1 interacts with PR-DPR. We constructed three deletion mutants of the amino acid sequence of the coding region of NOVA1. They are N1 (fragment comprising amino acids 1–169), N2 (fragment comprising amino acids 170–340), and N3 (fragment comprising amino acids 341–507) (Figure 3A). We used a yeast two-hybrid assay to determine whether the NOVA1 deletion mutant would affect the interaction with PR_50_. The results showed that deletion of the N3 region from NOVA1 abolished the interaction with PR_50_ (Figure 3B). Therefore, the N3 region of NOVA1 is required for interaction with PR_50_. Because the N3 region of NOVA1 contains the KH-1 functional domain, it is related to its activity of binding specific RNA sequences. Therefore, PR_50_ binding may specifically abolish the RNA-binding function of NOVA1 and inhibit its role as a splicing repressor for *UNC13A* CE.

### 3.4. CE Inclusion of UNC13A mRNA in Neuronal Cell Lines Will Be Significantly Increased by Knockdown of NOVA1, Resulting in Downregulation of UNC13A Protein Expression

**Figure 4:**
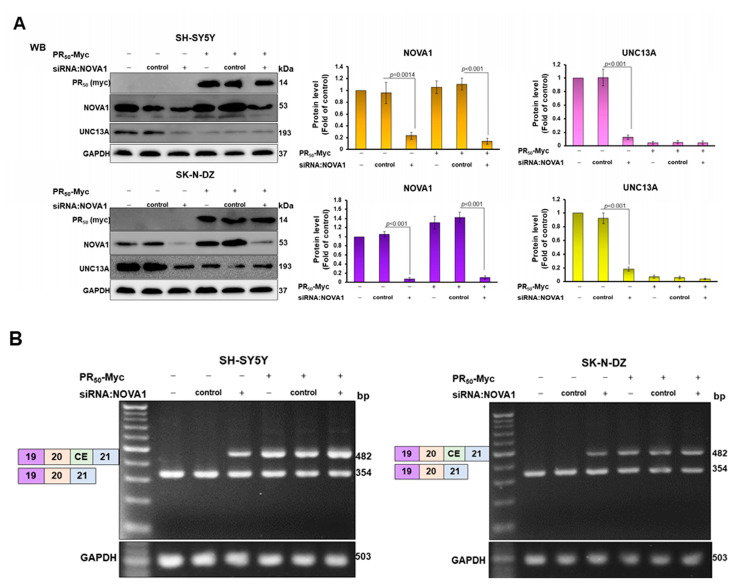
Knockdown of *NOVA1* enhances CE inclusion of *UNC13A* mRNA in SH-SY5Y and SK-N-DZ cell lines, leading to downregulation of UNC13A protein expression. SH-SY5Y or SK-N-DZ cell lines containing empty vectors or PR_50_ plasmids were transfected with NOVA1-specific or control non-specific siRNA. After 24 h of incubation, the level of UNC13 A protein and the CE inclusion of *UNC13A* mRNA were analyzed. (**A**) The expression of PR_50_ (Myc), NOVA1, and UNC13A was detected using Western blotting. (**B**) Analysis of CE inclusion in *UNC13A* mRNA using RT-PCR. The protein expression and DNA quantity used GAPDH as the internal loading control. In addition, the signal intensity was quantified using ImageJ software (version 1.53).

To investigate the role of NOVA1 in PR-DPR-induced CE inclusion of *UNC13 A* mRNA, we blocked NOVA1 expression in SH-SY5Y and SK-N-DZ cell lines using siRNA. After 24 h of treatment, in the NOVA1 siRNA group, the expression of NOVA1 decreased by 75.7% (*p* = 0.0014) and 92.7% (*p* < 0.001) in the SH-SY5Y and SK-N-DZ cell lines, respectively, compared with the control siRNA group by Western blot analysis (Figure 4A). RT-PCR analysis showed that knockdown of *NOVA1* promoted the increase in CE inclusion of *UNC13A* mRNA in both cell lines (Figure 4B), and finally resulted in the downregulation of UNC13A protein expression (*p* < 0.001, Figure 4A). Therefore, NOVA1 may be another splicing factor involved in CE inclusion of *UNC13A* mRNA besides TDP-43.

In the analysis of the PR_50_ expression group, after SH-SY5Y and SK-N-DZ cells were treated with *NOVA1* siRNA for 24 h, compared with the control siRNA group, the NOVA1 protein expression of the two cell lines decreased by 87.4% (*p* < 0.001) and 92.5% (*p* < 0.001), respectively (Figure 4A). The results of RT-PCR and Western blotting showed that, when PR_50_ expression and NOVA1 knockdown occurred simultaneously, CE inclusion of *UNC13A* mRNA (Figure 4B) and protein expression of UNC13A (Figure 4A) did not change significantly compared with the PR_50_ expression group. However, compared with the *NOVA1* siRNA group, the PR_50_ expression group had a stronger ability to induce CE inclusion of UNC13A mRNA (Figure 4B). Consequently, the downregulation of UNC13A protein expression was greater (Figure 4A). Based on the above results, we speculate that PD-DPR may cause CE inclusion of *UNC13A* mRNA and downregulation of protein expression partly by binding and inhibiting the activity of NOVA1.

### 3.5. Betulin (BT) Can Directly Interfere with the Interaction between PR-DPR and NOVA1 in the Yeast Two-Hybrid Model

**Figure 5:**
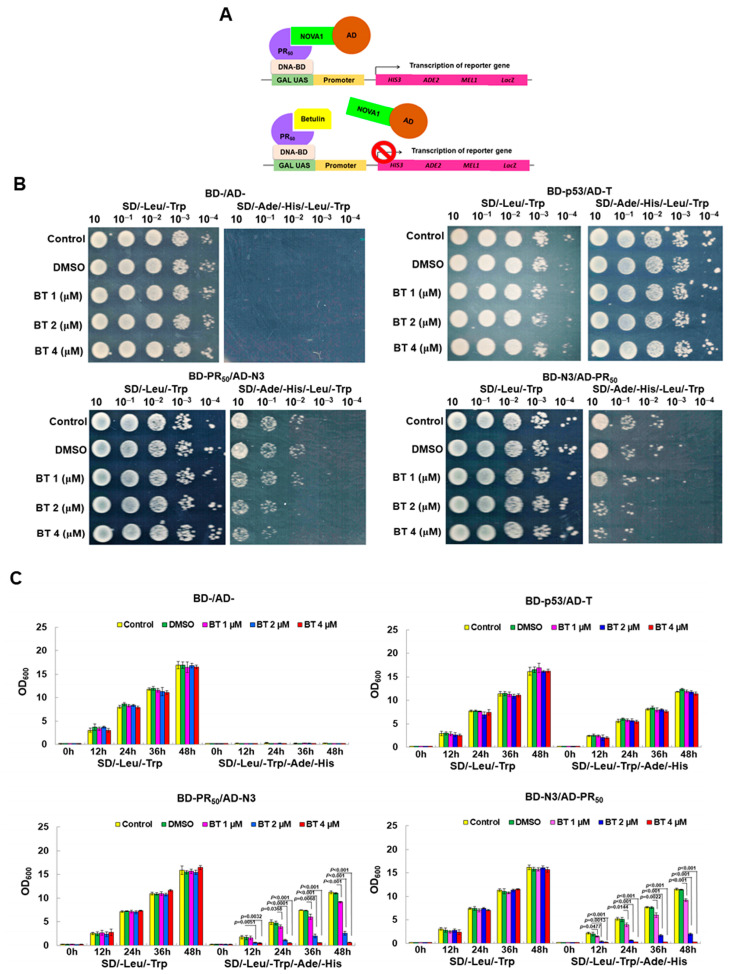
Betulin (BT) treatment abolishes the PR-DPR interaction with NOVA1 in a yeast two-hybrid model. (**A**) Schematic representation of the strategy of yeast two-hybrid-based growth assay for screening inhibitors of PR-DPR interaction with NOVA1. (**B**) In the yeast spot assay, yeast diploids of BD-/AD-, BD-p53/AD-T, BD-PR_50_/AD-N3(NOVA1), and BD-N3/AD-PR_50_ were cultured in non-selective borth (SD/-Leu/-Trp) containing serially diluted BT to logarithmic phase. The cultures were then normalized, serially diluted, and spotted on non-selective and selective (SD/Leu/-Trp/-Ade/-His) plates at 30 °C for 3 days. Finally, yeast growth was observed with the naked eye. (**C**) The yeast diploids were cultured to the logarithmic phase in non-selective broth containing serially diluted BT. The cultures were then normalized (OD_600_ = 0.2) and inoculated into selective and non-selective broth containing serially diluted BT for 2 days. The absorbance value was measured every 12 h.

On the basis of our findings that the interaction of PR-DPR and NOVA1 causes CE inclusion of *UNC13A* mRNA and decreased protein expression, we would like to establish a novel therapeutic strategy for C9-ALS targeting this abnormal interaction. It is relatively simple to screen and obtain candidate small molecules that can abrogate this interaction. We used the existing phytocompounds in the laboratory to perform growth analysis based on the yeast two-hybrid assay and used effective molecules to inhibit the growth characteristics of diploid yeast expressing PR_50_ and NOVA1 on selective media (Figure 5A). The results showed that BT has this remarkable property. Yeast spot assays on non-selective plates (SD/-Leu/-Trp) showed that diploid yeasts of BD-/AD-, BD-p53/AD-T, BD-PR50/AD-N3(NOVA1), and BD-N3/AD-PR_50_ could grow normally when BT treatment was lower than 4 μM (Figure 5B). This indicated that a treatment dose of BT below 4 μM did not affect the normal growth of yeast. On the selective plate (SD/-Ade/-Leu/-His/-Trp), the growth of diploid yeast of BD-p53/AD-T was not affected (Figure 5B, upper right), indicating that BT did not affect the interaction between p53 and T-antigen. In contrast, diploid yeasts of BD-PR_50_/AD-N3 and BD-N3/AD-PR_50_ showed dose-dependent growth inhibition of BT on selective plates (Figure 5B bottom). To quantify yeast two-hybrid-based growth assays, we cultured diploid yeast in broth and performed absorbance (OD_600_) measurements at the indicated time points (Figure 5C). The results showed that, after 48 h of culture, there was no significant difference in the growth rate between the 4 μM BT group and the DMSO group in the non-selective broth. In selective broth, the growth rates of diploid yeasts of BD-PR_50_/AD-N3 and BD-N3/AD-PR_50_ were reduced by 94.9% (*p* < 0.001, bottom left, Figure 5C) and 97.6% (*p* < 0.001, bottom right, Figure 5C). In contrast, diploid yeast of BD-p53/AD-T showed no change in growth (top right, Figure 5C). This indicated that BT treatment could effectively and specifically inhibit the interaction between PR_50_ and NOVA1.

### 3.6. BT treatment Can Significantly Inhibit the CE Inclusion of UNC13 A mRNA in Neuronal Cell Lines to Avoid Downregulation of UNC13 A Protein Expression

**Figure 6:**
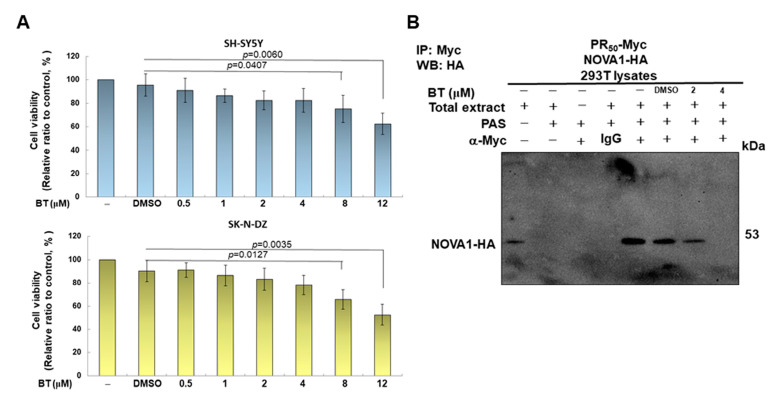

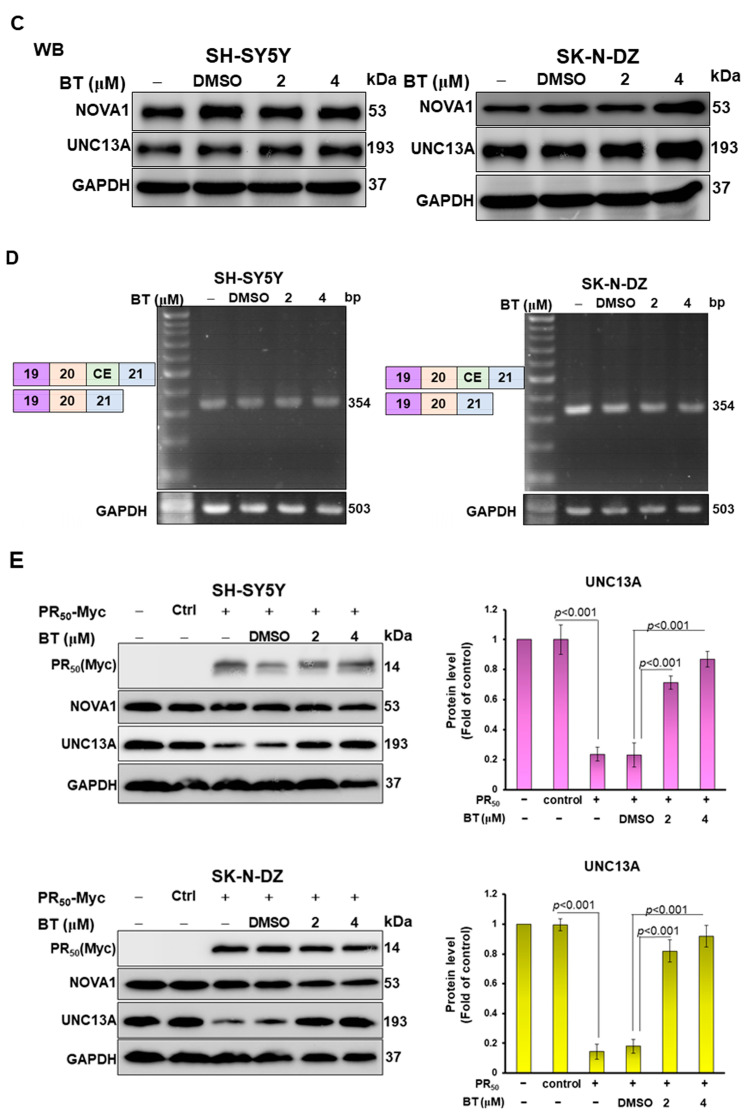

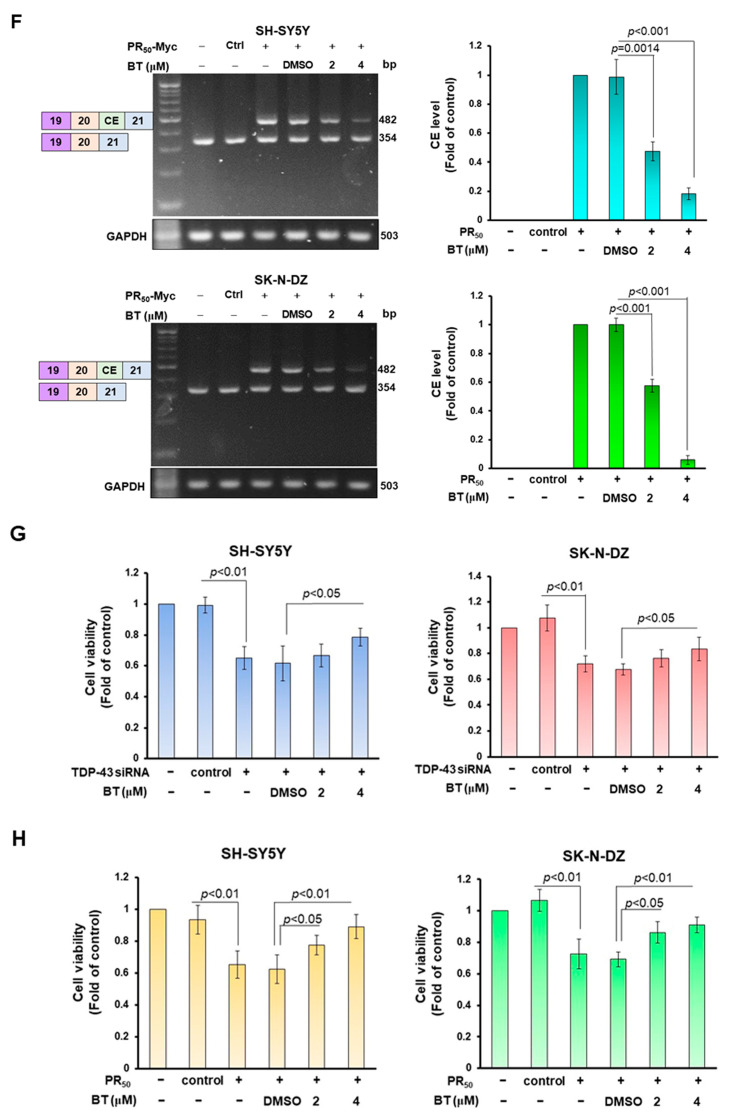
PR-DPR-induced CE inclusion of *UNC13A* mRNA in SH-SY5Y or SK-N-DZ cell lines and the resulting downregulation of UNC13A protein expression can be reversed by BT treatment. (**A**) SH-SY5Y and SK-N-DZ cells were treated with a series of BT concentrations for 24 h and then their cell viability determined using CellTiter-Blue^®^ Reagent. The results show that BT does not affect cell survival at concentrations below 4 μM. (**B**) BT inhibits the interaction between PR_50_ and NOVA1 in 293T cells. Lysates of 293T cells coexpressing PR_50_-Myc and NOVA1-HA were added to 2 or 4 μM BT and coimmunoprecipitated with Myc antibody. Finally, Western blotting was performed using an HA antibody. (**C**) Western blot analysis showed no change in the expression of NOVA1 and UNC13A in SH-SY5Y and SK-N-DZ cells after BT treatment. (**D**) RT-PCR showed that the CE inclusion of *UNC13A* mRNA in SH-SY5Y and SK-N-DZ cells did not change after BT treatment. (**E**) Western blotting showed that the expression of NOVA1 was unchanged but the expression of UNC13A was increased in SH-SY5Y and SK-N-DZ cells expressing PR_50_ after BT treatment. (**F**) RT-PCR analysis showed that PR_50_-expressing SH-SY5Y and SK-N-DZ cells were significantly reduced in CE inclusion of *UNC13A* mRNA after BT treatment. GAPDH was used as the internal loading control for above experiments. (**G**,**H**) Using MTT assay to evaluate the effect of BP on the survival of *TDP-43* knockdown cells (**G**) and PR_50_-overexpressing cells (**H**).

We showed in previous experiments that the interaction between PR-DPR and NOVA1 can be disrupted by BT treatment in a yeast two-hybrid model. Next, we aimed to assess whether BT treatment could reverse PR_50_-induced CE inclusion of *UNC13A* mRNA and downregulation of protein expression in SH-SY5Y and SK-N-DZ cell lines in vitro. First, we used CellTiter-Blue^®^ Reagent to detect the viability of SH-SY5Y and SK-N-DZ cells treated with a series of BT concentrations to determine the appropriate BT concentration. We found that the survival rate of the cells did not significantly change when the BT concentration was less than 4 μM (Figure 6A). Therefore, we used BT up to 4 μM for follow-up studies. We also demonstrated that BT concentration-dependently reduced the interaction between PR_50_ and NOVA1 in 293T cell lysates using co-immunoprecipitation (Figure 6B). Western blot analysis showed that BT did not affect the expression of NOVA1 and UNC13A in SH-SY5Y and SK-N-DZ cells (Figure 6C). Likewise, BT did not affect the CE inclusion of *UNC13A* mRNA as analyzed by RT-PCR (Figure 6D). Next, we analyzed the effect of BT on PR_50_-expressing cells. Western blot analysis showed that BT concentration-dependently increased the expression of the UNC13A protein in cells (Figure 6E). Under 4 μM BT, compared with the DMSO group, the expression of UNC13A increased by 3.8-fold (*p* < 0.001) and 5.1-fold (*p* < 0.001) in SH-SY5Y and SK-N-DZ cells expressing PR_50_, respectively (Figure 6E). RT-PCR showed that the CE inclusion of *UNC13A* mRNA in cells decreased in a BT concentration-dependent manner (Figure 6F). Under 4 μM BT, compared with the DMSO group, CE inclusion of *UNC13A* mRNA was reduced by 81.5% (*p* < 0.001) and 94.0% (*p* < 0.001) in SH-SY5Y and SK-N-DZ cells expressing PR_50_, respectively (Figure 6F). From these results, it can be confirmed that BT can effectively inhibit the CE inclusion of *UNC13A* mRNA caused by the expression of PR-DPR in neuronal cell lines, thus restoring the expression of the UNC13A protein. Finally, we analyzed the effects of BT on survival rate of TDP-43 knockdown and PR_50_-expressing cells. The results showed that 4 μM BP treatment could improve the survival rate of TDP-43-expressing SH-SY5Y (*p* < 0.05) and SK-N-DZ (*p* < 0.05) cells (Figure 6G) as well as PR_50_-expressing SH-SY5Y (*p* < 0.01) and SK-N-DZ (*p* < 0.01) cells (Figure 6H).

## 4. Discussion

Many human genes are post-transcriptionally modified by alternative splicing (AS) to regulate mature mRNA stability, translation efficiency, protein diversity, and/or NMD [33]. The appropriate temporal and spatial expression of AS is closely related to biological processes such as cell development, differentiation, and functional survival [34]. Multiple RNA-binding proteins (RBPs) are master regulators of AS. These RBPs can be classified as activators or repressors of specific exons. They bind pre-mRNAs to control the inclusion or exclusion of partial sequences (exons or a small part of introns) of nascent transcripts into mature mRNAs. Increasing reports have shown that RBP-mediated dysregulation of AS plays an important pathogenic role in various neurodegenerative diseases [35].

TDP-43 is an RNA-binding protein that functions as a splicing repressor. Previous studies have shown that loss of function or cytoplasmic translocation of TDP-43 is closely associated with ALS [31,32]. Recent studies have shown that these defects cause cryptic exon (CE) inclusion of the mRNA of *UNC13A*, an important gene for presynaptic release of neurotransmitters, and ultimately lead to NMD-induced downregulation of UNC13A protein expression. Studies have shown that this result is also partially observed in patients with C9-ALS. Therefore, we believe that some of the pathological features of C9-ALS may be related to CE inclusion of *UNC13A* mRNA. The production of PR-DPR has been considered as an important gain-of-function pathogenic factor in C9-ALS at the cellular level. The purpose of this study was to investigate the association between C9-ALS-associated PR-DPR and CE inclusion of *UNC13A* mRNA in neurons. The results showed that PR-DPR significantly induced CE inclusion of *UNC13A* mRNA and downregulated UNC13A protein expression in neural cell lines. This is the first study to demonstrate that PR-DPR in C9-ALS promotes CE inclusion of *UNC13A* mRNA and thus reduces UNC13A protein expression.

PR-DPR occurs mainly in the nucleus and affects the function of many proteins in the nucleus, including various splicing factors [6]. The literature shows that reduction in expression, loss of function, or translocation of TDP-43 has been observed in C9-ALS models [36]. Therefore, it was expected that PR-DPR would affect TDP-43 to promote CE inclusion of UNC13A mRNA, leading to decreased protein expression. However, when we performed the yeast two-hybrid assay of PR-DPR and TDP-43 protein, no interaction was found. This suggests that other pathways contribute to the dysregulation of TDP-43 in C9-ALS. To confirm the possible mechanism of PR-DPR promoting CE inclusion of UNC13A mRNA, we used PR-DPR as bait to perform yeast two-hybrid screening. NOVA1 was identified as an interaction partner in positive clones. NOVA belongs to the neuron-specific KH-type RBP protein family, whose members include NOVA1 and NOVA2. It can combine with YCAY motifs to participate in AS of specific genes [29]. NOVA1 is closely related to the survival and development of neurons and is involved in AS regulation of neurotransmitter receptors such as GABAAγ2 and GlyRα2 [37]. NOVA1 knockout mice show progressive motor deficits and premature death due to apoptosis of spinal cord and brainstem neurons [38]. In the spinal muscular atrophy (SMA) mouse model, NOVA1 expression and survival of motor neuron 2 (SMN2) were synchronously downregulated. This is because NOVA1 acts on exon 7 of SMN2, and the lack of NOVA1 reduces the inclusion of exon 7 in SMN2, thereby reducing the expression of the SMN protein [39]. Moreover, NOVA1 binds to the mRNA of a specific gene mainly in introns and is associated with CE splicing. This triggers NMD to regulate neuronal homeostasis, such as excitation/inhibition balance [40]. Furthermore, the genes mainly regulated by NOVA1 in the brain are related to axon guidance and synapses, such as channels, neurotransmitter receptors, scaffolds, and adhesion proteins [41]. It is worth noting that Krach et al. found high levels of NOVA1 in the insoluble protein fraction of induced pluripotent stem-cell-derived motor neurons (MN) from patients with sporadic and familial ALS, and the levels of NOVA1 in the nucleus were reduced. It was also observed that AS events in ALS-associated MN are enriched in the binding site of NOVA1 [30]. To confirm the role of NOVA1 in CE inclusion of *UNC13A* mRNA, we blocked the expression of NOVA1 using siRNA and found that CE inclusion of *UNC13A* mRNA increased and protein expression decreased. In addition, knockdown of NOVA1 did not increase the ability of PR_50_ to induce CE inclusion of *UNC13A* mRNA. This illustrates that PR_50_ regulates the CE inclusion of *UNC13A* mRNA in part by inhibiting the function of NOVA1. Therefore, NOVA1 may play a role similar to that of TPD-43.

In this study, we were also interested in how NOVA1 regulates the CE of *UNC13A* mRNA. Where is its binding site on mRNA? Whether it is associated with the ALS-related SNP site on the *UNC13A* gene is another question. We used RBPmap (https://rbpmap.technion.ac.il/ (accessed on 20 March 2023)) [42] for analysis and found that there are six possible NOVA1 binding sites (UUCAUAA or AUCAC) in the intron sequence between exon 20 and exon 21 of *UNC13A* pre-mRNA. These loci are not on the ALS-associated SNP of the *UNC13A* gene. In the future, we will use RNA co-immunoprecipitation and site-directed mutagenesis to confirm the binding of NOVA1 at these six predicted sites.

Finally, we found that betulin (BT), a lupine-type pentacyclic triterpenoid present in the bark of birch, blocks the interaction of PR-DPR with NOVA1, reverses the CE inclusion of *UNC13A* mRNA, and avoids downregulation of protein expression. BT has anti-oxidative, anti-inflammatory, anti-amyloid accumulation, and neuroprotective functions [43]. Therefore, BT treatment can not only improve the expression of UNC13A and restore the release function of neurotransmitters in the synapses of motor neurons but also improve the oxidative stress of nerve cells and the microenvironment of chronic inflammation caused by various environmental or genetic factors.

## 5. Conclusions

In summary, this study provides a possible mechanism for the CE inclusion of *UNC13A* mRNA and the decrease in protein expression in C9-ALS patients. This at least partially caused by C9-ALS-associated PR-DPR inhibiting the splicing modulating function of NOVA1. In addition, we also found that the phytocompound BT can abolish the interaction between PR-DPR and NOVA1, which can improve the protein expression of UNC13A by reducing CE inclusion of mRNA. In the future, we can further use the motor neuron model derived from induced pluripotent stem cells of C9-ALS patients to evaluate the potential of BT for treating C9-ALS.

## Data Availability

All data used and analyzed during the current study are available from the corresponding author upon reasonable request.
